# Influence of kidney replacement therapy on indirect calorimetry in critically ill patients

**DOI:** 10.1038/s41430-025-01643-9

**Published:** 2025-07-30

**Authors:** Annalena Knoll, Sirak Petros, Bastian Pasieka, Lorenz Weidhase

**Affiliations:** University Hospital Leipzig, Medical ICU, Leipzig, Germany

**Keywords:** Translational research, Metabolism

## Abstract

**Background:**

Kidney replacement therapy (KRT) is frequently implemented in the intensive care unit. While measuring energy expenditure is recommended in the critically ill, the influence of KRT on indirect calorimetry (IC) is not fully clear. This prospective study aimed to investigate the influence of continuous veno-venous hemodialysis (CVVHD) and slow extended daily dialysis (SLEDD) on IC variables.

**Patients and methods:**

We included critically ill mechanically ventilated adult medical patients on KRT for acute kidney injury. CVVHD was run with regional citrate anticoagulation, while SLEDD with systemic heparin anticoagulation. We conducted IC twice on every patient, either immediately before the planned termination of KRT and then an hour after the end of KRT or immediately before commencement of KRT and then again after an hour on KRT.

**Results:**

We included 100 patients (75 males) with a median age of 64.0 years, a mean APACHE-II score of 30.9 and a mean SOFA score of 11.3 on the day of IC. There was no significant difference in median resting energy expenditure with versus without CVVHD (8029 [6993–9644] versus 7814 [6962–9304] kJ, *p* = 0.75) as well as with versus without SLEDD (9258 [8017–10,364] versus 9269 [8070–11,065] kJ, *p* = 0.63). The difference in resting energy expenditure between the two measurements was also not significant regardless of the sequence of IC measurements (*p* = 0.69).

**Conclusion:**

This prospective study on critically ill adult patients did not show any significant difference for indirect calorimetry variables between measurements conducted during CVVHD and SLEDD compared to those without KRT. ClinicalTrials.gov ID: NCT04599569

## Introduction

The optimal energy provision to the critically ill patient is variable, depending on the course of the acute disease condition [1]. While hypocaloric nutrition is favored during the initial phase of the acute illness, a gradual increase in energy provision approaching normocaloric feeding is suggested during the late phase of the acute illness and rehabilitation. International guidelines recommend that energy expenditure of this patient population, particularly those on mechanical ventilation, should be measured using indirect calorimetry (IC) [1, 2].

Acute kidney injury (AKI) is common among critically ill patients [3], and a large proportion of this population is also on mechanical ventilation [4]. Kidney replacement therapy (KRT) is frequently implemented among this patient population [5]. It is not fully clear whether KRT may have a clinically relevant impact on IC. Resting energy expenditure with IC is calculated by means of the de Weir formula using oxygen consumption (VO_2_) and carbon dioxide production (VCO_2_). However, particularly VCO_2_ measurement may be affected due to gas exchange across the dialysis membrane, although its extent is not certain [6]. Physiologically, the combination of CO_2_ with water in the red blood cells in the presence of carbonic anhydrase accounts for the majority of the CO_2_ transport, followed by a far less portion transported as carbamino combination with hemoglobin and other plasma proteins, and only a small portion of the CO_2_ transported in the dissolved state [7]. Therefore, it is difficult to determine the gas exchange during KRT. One old experimental study reported that a considerable amount of CO_2_ may be eliminated during acetate maintenance hemodialysis [8]. Another study on patients on maintenance hemodialysis showed excess CO_2_ generation during high efficiency bicarbonate hemodialysis [9]. An experimental study on stable chronic hemodialysis patients reported an O_2_ and CO_2_ transfer from the dialysate space into the blood after 5 min of high-flux hemodialysis [10]. A recent pilot study concluded that a relevant amount of CO_2_ can be removed with continuous veno-venous hemofiltration (CVVH) [11]. However, its effect on indirect calorimetry is not fully elucidated. One prospective observational study did not demonstrate a significant difference in energy expenditure with versus without KRT [12]. However, that study included predominantly high-volume peritoneal dialysis and conventional hemodialysis, which are not commonly implemented in critically ill patients with AKI.

The primary aim of the present observational study is to evaluate whether KRT has got any significant effect on IC variables in critically ill mechanically ventilated patients managed with KRT for AKI.

## Patients and methods

The present study is a prospective observational study on critically ill mechanically ventilated adult medical patients admitted to the Medical ICU of the University Hospital Leipzig, Germany, and managed with KRT for acute renal failure. The study was approved by the institutional review board (file number 338/20-ek), and it was conducted in accordance with the Declaration of Helsinki for experiments involving humans. The patients were included after informed consent of the patient or a legal guardian. This is an exploratory study, as sample size calculation could not be done due to the lack of background data.

Inclusion criteria were invasive mechanical ventilation and KRT either already established or planned to start for acute kidney injury. Exclusion criteria were a positive end-expiratory pressure (PEEP) > 16 cm H_2_O, thorax drain for pneumothorax or any air leak in the ventilator system, age <18 years, or lack of consent. A blood lactate level of ≥4 mmol/L (measured with the blood gas analyzer ABL Flex 800, Radiometer, Krefeld, Germany) was considered as a sign of metabolic instability, so that such patients were also excluded.

IC was conducted according to widely accepted standard procedures with the Cosmed QUARK RMR (Cosmed, Rome, Italy). IC calibration was performed before every measurement. Ventilator variables were left unchanged starting at least 30 min ahead of and until the end of the IC. Sedation, analgesia, vasopressor support and artificial nutrition were left unchanged during the IC measurements. The study patients were also left undisturbed during the IC. Every IC measurement lasted at least 35 min, with the readings of the first 5 min discarded, so that IC records of at least 30 min were available for every IC run. If a manipulation had to be done on the patient for clinical reasons, the IC measurement was excluded from further analysis.

CVVHD with regional citrate anticoagulation (RCA) and SLEDD with systemic anticoagulation with unfractionated heparin are the standard KRT procedures in the unit, with the choice left to the discretion of the responsible physician. Intensivists not involved in the study were responsible for the decision on the type and time point of KRT commencement and termination. The sequence of IC measurements was then adapted based on the clinical decision.

CVVHD was carried out using the Multifiltrate System and SLEDD with the Genius® 90 Therapy System (both from Fresenius Medical Care, Bad Homburg, Germany). The dialysate for CVVHD contained 20.0 mmol/L bicarbonate (in order to reduce the expected metabolism of citrate to bicarbonate), while this was 35.0 mmol/L for SLEDD according to the manufacturer’s specifications. Trinatrium citrate was used for RCA during CVVHD. To avoid heat loss and its effect on energy expenditure, we adjusted circuit temperature for CVVHD at 38 °C, and the dialysate was prewarmed for SLEDD according to manufacturer’s instructions. In both modalities, a high-flux dialyzer was used (for CVVHD Ultraflux AV 1000S^®^ with 1.8 m^2^ effective surface area; for SLEDD Genius^®^ sleddFlux with 0.7 m^2^ effective surface area, both from Fresenius Medical Care, Bad Homburg, Germany).

We have conducted IC twice on every patient, either immediately before termination of the KRT and then an hour thereafter (scenario 1) or immediately before the start of KRT followed by a second IC an hour on KRT (scenario 2). In both scenarios, we performed the second IC run an hour after the patients were in the corresponding state (either following the termination of KRT or an hour after the KRT was started). This period was considered adequate to account for a steady state.

We have collected data for age, gender, actual body weight, ideal body weight (using the Hamwi equation [13]) and body mass index (BMI). In patients with a BMI > 30, we calculated adjusted body weight using the following equation: adjusted body weight = ideal body weight + 0.4(actual body weight − ideal body weight). Furthermore, the data for Acute Physiology And Chronic Health (APACHE)-II score on ICU admission, the Sequential Organ Function Assessment (SOFA) score on the study day, mode of mechanical ventilation, mode of KRT, including blood and dialysate flow rates, vasopressor requirement, mean arterial blood pressure, body temperature (taken via indwelling urinary bladder catheter with integrated temperature sensor) during both IC runs, room temperature and room humidity. Regarding the IC, energy expenditure, oxygen consumption (VO_2_), CO_2_ production (VCO_2_) and respiratory quotient (RQ) were recorded.

Statistical analysis was conducted with SPSS for Windows Version 29 and GraphPad Prism Version 10. Categorical variables are given as percentiles and analyzed with the chi square test or Fisher exact test as appropriate. Metric variables are reported as mean with standard deviation or median with 25 and 75 quartiles in square brackets depending on their normal distribution according to the Shapiro-Wilk test. Following that, comparisons of data with versus without KRT were performed either with the paired Student t test or Wilcoxon signed rank test as appropriate. Furthermore, we have also looked whether the sequence of IC run, that is scenario 1 vs. scenario 2, would have any impact on energy expenditure measurements. For this purpose, we compared the two scenarios based on the differences between the variables of the two IC runs. Due to the organizational structure in the implementation of SLEDD, this comparison of scenarios was possible only with CVVHD. A two-tailed *p* value < 0.05 is considered statistically significant.

## Results

We have included 100 critically adult mechanically ventilated patients. During the indirect calorimetry, 91 patients were on pressure-assisted mode, while only nine patients were on volume-controlled mode. KRT was carried out with CVVHD in 77 patients and with SLEDD in 23 patients. Demographic and clinical characteristics are given in Table 1. The median venous blood lactate of the CVVHD cohort was higher than that of the SLEDD cohort (1.5 [1.2–2.2] vs. 1.0 [0.8–1.5] mmol/L, *p* = 0.003).

**Table 1:** Demographic and clinical characteristics of the study population.

Variable	Total cohort (*N* = 100)	CVVHD (*N* = 77)	SLEDD (N23)	p(CVVHD vs. SLEDD)
Age (years)	64.0 [55.3–74.0]	64.0 [55.0–74.0]	65.0 [56.0–76.0]	0.67
Males (%)	75.0	71.4	87.0	0.17
Actual body weight (kg)	92.0 ± 24.5	91.9 ± 23.3	92.3 ± 28.6	0.94
Ideal or adjusted body weight (kg)	77.7 ± 14.2	76.9 ± 13.7	80.3 ± 15.8	0.32
APACHE-II score	30.9 ± 8.9	30.5 ± 8.5	32.1 ± 10.0	0.44
SOFA score on IC day	11.3 ± 3.8	11.8 ± 3.9	9.5 ± 2.4	0.004
Vasopressor infusion during IC (%)	61.0	68.8	34.8	0.006
MAP during IC (mm Hg)	75.0 [68.0–82.0]	76.0 [66.5–83.5]	72.0 [64.0–82.0]	0.39
Continuous sedation during IC (%)	75.0	84.4	43.5	<0.001
Body temperature during IC (°C)	37.1 ± 0.8	37.0 ± 0.8	37.2 ± 0.7	0.16
Room temperature (°C)	23.0 ± 1.1	23.0 ± 1.0	23.2 ± 1.1	0.49
Humidity (%)	37.5 ± 10.4	37.8 ± 10.1	36.5 ± 11.7	0.62

*BMI* body mass index, *APACHE* Acute Physiology And Chronic Health, *IC* indirect calorimetry, *MAP* mean arterial pressure, *SOFA* Sequential Organ Function Assessment.

While 72 patients were on enteral nutrition prior as well as during the IC, 16 patients were on parenteral nutrition, seven patients received enteral nutrition with supplemental parenteral nutrition, only five patients were not receiving any feeding.

The median blood flow rate was 100 [100–120] ml/min during CVVHD and 150 [150–170] ml/min during SLEDD. The median dialysate flow rate was 2000 [2000–2500] ml/h during CVVD and 9000 [9000–10,200] ml/h during SLEDD. The pH of the patients at the beginning of the hemodialysis was 7.35 ± 0.7 in the CVVHD cohort and 7.34 ± 0.06 in the SLEDD cohort (*p* = 0.69)

The body temperature of the patients was only slightly lower during CVVHD, even though statistically significant, while there was no significant difference in mean arterial pressure at any time. There was no significant difference between IC data measured during KRT and those without KRT for both CVVHD and SLEDD (Table 2).

**Table 2:** Data for indirect calorimetry variables, body temperature and mean arterial pressure with and without kidney replacement therapy (KRT).

Variable	With KRT	Without KRT	*p*
CVVHD			
Body temperature (°C)	37.0 ± 0.8	37.2 ± 0.8	0.01
MAP (mm Hg)	76.0 [66.5–83.5]	75.0 [67.0–84.0]	0.86
REE kJ/d	8029 [6993–9644]	7814 [6962–9304]	0.75
kJ/kg IBW	108.4 [89.5–128.0]	105.2 [90.8–125.8]	
kcal/d	1918 [1671–2304]	1867 [1663–2223]	
kcal/kg IBW	25.9 [21.4–30.6]	25.1 [21.7–30.1]	
RQ	0.75 [0.69–0.79]	0.76 [0.71–0.80]	0.23
VO_2_ (ml/min)	281.0 [243.7–338.3]	272.2 [245.4–332.5]	0.72
VCO_2_ (ml/min)	214.6 [179.0–252.6]	217.0 [176.0–245.0]	0.41
SLEDD			
Body temperature (°C)	37.2 ± 0.7	37.3 ± 0.8	0.25
MAP (mm Hg)	72.0 [64.0–82.0]	74.0 [67.0–82.0]	0.73
REE kJ/d	9258 [8017–10364]	9269 [8070–11065]	0.63
kJ/kg IBW	119.1 [92.3–134.3]	121.7 [96.7–133.1]	
kcal/d	2212 [1938–2304]	2214 [1928–2223]	
kcal/kg IBW	28.5 [22.0–32.1]	29.1 [23.1–31.8]	
RQ	0.75 [0.71–0.79]	0.74 [0.72–0.80]	0.16
VO_2_ (ml/min)	324.7 [282.3–338.3]	327.9 [281.4–332.5]	0.48
VCO_2_ (ml/min)	240.9 [205.1–252.6]	254.1 [219.5–245.0.]	0.81

*CVVHD* continuous veno-venous hemodialysis, *SLEDD* slow extended daily dialysis, *MAP* mean arterial pressure, *REE* resting energy expenditure reported in kilojoules (kJ) and kilocalories (kcal), *IBW* ideal or adjusted body weight, *kg* kilogram, *RQ* respiratory quotient, *VO*_2_ oxygen consumption, *VCO*_2_ CO_2_ production.

Regarding the IC scenario, 32 (all of them with CVVHD) runs were conducted as scenario 1 and 68 (45 with CVVHD and 23 with SLEDD) as scenario 2. Since all patients with SLEDD were in scenario 2, we conducted the comparisons between scenario 1 and 2 only for those with CVVHD. We did not detect any significant difference between the two scenarios regarding changes in IC variables (Table 3).

**Table 3:** Differences between indirect calorimetry variables with versus without CVVHD based on the scenario of indirect calorimetry runs.

Variable	Scenario 1 (*N* = 32)*	Scenario 2 (*N* = 45)^a^	*P*
Delta REE			
kJ/d	36 [−759–450]	−95 [−517–565]	0.69
(kcal/d)	(8.5 [−181–107])	(−23 [−124–135])	
Delta RQ	−0.02 [−0.04–0.01]	0.00 [−0.03–0.03]	0.14
Delta VO_2_ (ml/min)	1.7 [−26.5–15.3]	3.2 [−18.8–19.7]	0.71
Delta VCO_2_ (ml/min)	−4.8 [−20.0–17.8]	4.5 [−13.2–12.5]	0.39

*REE* resting energy expenditure, *RQ* respiratory quotient, *VO*_2_ oxygen consumption, *VCO*_2_ CO_2_ production.

^a^Scenario 1: Indirect calorimetry first with and then without CVVHD; scenario 2: indirect calorimetry first without and then with CVVHD.

## Discussion

This prospective study on critically ill adult medical patients on mechanical ventilation did not show any significant difference for indirect calorimetry variables between measurements conducted during CVVHD with RCA and SLEDD with systemic heparin anticoagulation compared to those without KRT. A previous observational study reported similar findings. However, that study included patients on conventional hemodialysis and peritoneal dialysis, which are not commonly implemented in critically ill patients with acute kidney injury [12]. We have included critically ill patients managed with the two KRT modalities widely implemented in the ICU, namely CVVHD and SLEDD.

The MECCIAS trial conducted in ten critically patients showed that CO_2_ is removed during CVVH with regional citrate anticoagulation. However, the authors concluded that this was not clinically significant so that a correction factor for REE was not required [14]. Although this supports our findings, one should be careful to extrapolate conclusions without due consideration of the modality, type of dialysate and setting of the KRT. The Weir equation applied during IC assumes that VO_2_ and VCO_2_ are the results of energy production. Therefore, depending on the extent, a variable CO_2_ movement along the dialysis membrane during KRT may have an impact on the basic principle of indirect calorimetry. During hemodialysis with regional citrate anticoagulation, the bicarbonate administered directly via the dialysate as well as indirectly through citrate metabolism in the liver might lead to a rise in VCO_2_. Therefore, the possible loss of CO_2_ through the dialysis membrane may be compensated through this CO_2_ gain. In one older study, bicarbonate infusion resulted in a temporary rise in VCO_2_, with this effect almost lost 15 min after the end of the infusion [15].

Heat loss during KRT can affect energy metabolism. However, sedation commonly administered during mechanical ventilation may blunt compensatory mechanisms [6]. Furthermore, the risk of hypothermia is low with modern KRT systems [16]. In this study, heat loss through the extracorporeal circuit during CVVHD was reduced by adjusting the circuit temperature. Regarding SLEDD, the hemodialysis was started immediately after the preparation of the system with the pre-warmed dialysate to minimize heat loss. Nevertheless, a certain degree of heat loss cannot be ruled out.

There is a net citrate uptake during KRT with RCA. Observational studies using CVVH with RCA have reported a net energy gain of 196–218 kcal/day due to the citrate uptake [17, 18]. Different CVVH settings result in different bioenergetic balances depending on the substitution fluids prescribed [19]. However, the dose of citrate during hemodialysis is lower than that during hemofiltration [20]. A study using CVVHD with RCA concluded that the net caloric gain through citrate uptake was only 135 kcal/day [21]. Therefore, the citrate gain during CVVHD in our study may not have had a significant impact on the indirect calorimetry. Furthermore, the literature findings regarding energy gain through citrate uptake assume that citrate would be immediately metabolized fully through the Krebs cycle into energy, which has not been confirmed in critically ill patients. Citrate may pass through diverse metabolic pathways [6]. Additionally, mitochondrial function may be impaired to a varying degree in critically ill patients [22, 23].

We did not find any significant difference regarding the sequence of the IC runs for CVVHD. Thus, filter life span did not seem to have a clinically relevant impact on the measurement if the prescribed filter life span is observed.

In conclusion, we did not observe a clinically significant difference in indirect calorimetry measurements with versus without CVVHD with RCA as well as SLEDD with systemic heparin anticoagulation in critically ill adult mechanically ventilated patients. Our findings may thus help to simplify IC handling under similar conditions. Nevertheless, due to the various modes and flow rates during KRT, the type of dialysate used and the extent of heat loss, caregivers should plan the IC runs on individual basis [14, 24].

Our study has certain limitations. First, we have included only two modes of KRT, so that the conclusions cannot be extrapolated to other modalities. Nevertheless, CVVHD and SLEDD are frequently applied modes in critically ill patients with acute kidney injury [25]. Second, we did not quantify the possible net energy gain following citrate uptake during CVVHD with RCA. However, previous studies have demonstrated that this gain is not high enough to have a significant impact on the measurement of energy expenditure. Third, we have applied a steady state period of an hour before the second IC measurement. It is not clear whether this period is enough to achieve a steady state before conducting the second IC. However, a longer waiting period would have the risk of incomparable steady states and it would have been difficult to implement in the ICU atmosphere. Therefore, our results should be interpreted with due consideration of the clinical condition of the patient and the prescribed mode and adjustments of the KRT.

## Data Availability

The data generated during this study are available from the corresponding author on reasonable request.

## References

[1] Singer P, Blaser AR, Berger MM, Alhazzani W, Calder PC, Casaer MP, et al. ESPEN guideline on clinical nutrition in the intensive care unit. Clin Nutr. 2019;38:48–79. 30348463 10.1016/j.clnu.2018.08.037

[2] McClave SA, Taylor BE, Martindale RG, Warren MM, Johnson DR, Braunschweig C, et al. Guidelines for the provision and assessment of nutrition support therapy in the adult critically ill patient: society of critical care medicine (SCCM) and American Society for Parenteral and Enteral Nutrition (A.S.P.E.N.). JPEN J Parenter Enter Nutr. 2016;40:159–211. 10.1177/0148607115621863 26773077

[3] Rachoin JS, Weisberg LS. Renal replacement therapy in the ICU. Crit Care Med. 2019;47:715–21. 30768442 10.1097/CCM.0000000000003701

[4] Bellomo R, Cass A, Cole L, Finfer S, Gallagher M, Lee J, et al. Calorie intake and patient outcomes in severe acute kidney injury: findings from The Randomized Evaluation of Normal vs. Augmented Level of Replacement Therapy (RENAL) study trial. Crit Care. 2014;18:R45. 24629036 10.1186/cc13767 PMC4057152

[5] Hoste EA, Bagshaw SM, Bellomo R, Cely CM, Colman R, Cruz DN, et al. Epidemiology of acute kidney injury in critically ill patients: the multinational AKI-EPI study. Intensive Care Med. 2015;41:1411–23. 26162677 10.1007/s00134-015-3934-7

[6] Jonckheer J, Spapen H, Malbrain M, Oschima T, De Waele E. Energy expenditure and caloric targets during continuous renal replacement therapy under regional citrate anticoagulation. A viewpoint. Clin Nutr. 2020;39:353–7. 30852030 10.1016/j.clnu.2019.02.034

[7] Hall JE. Transport of oxygen and carbon dioxide in blood and tissue fluids. In: GUYTON and HALL textbook of medical physiology. Philadelphia, USA: Elsevier; 2016. pp 527–37.

[8] Bosch JP, Glabman S, Moutoussis G, Belledonne M, von Albertini B, Kahn T. Carbon dioxide removal in acetate hemodialysis: effects on acid base balance. Kidney Int. 1984;25:830–7. 6433099 10.1038/ki.1984.97

[9] Symreng T, Flanigan MJ, Lim VS. Ventilatory and metabolic changes during high efficiency hemodialysis. Kidney Int. 1992;41:1064–9. 1513087 10.1038/ki.1992.162

[10] Sombolos KI, Bamichas GI, Christidou FN, Gionanlis LD, Karagianni AC, Anagnostopoulos TC, et al. pO_2_ and pCO_2_ increment in post-dialyzer blood: the role of dialysate. Artif Organs. 2005;29:892–8. 16266303 10.1111/j.1525-1594.2005.00126.x

[11] Jonckheer J, Spapen H, Debain A, Demol J, Diltoer M, Costa O, et al. CO_2_ and O_2_ removal during continuous veno-venous hemofiltration: a pilot study. BMC Nephrol. 2019;20:222. 31208356 10.1186/s12882-019-1378-y PMC6580471

[12] de Góes CR, Vogt BP, Sanches ACS, Balbi AL, Ponce D. Influence of different dialysis modalities in the measurement of resting energy expenditure in patients with acute kidney injury in ICU. Clin Nutr. 2017;36:1170–4. 27595381 10.1016/j.clnu.2016.08.008

[13] Hamwi GJ. Therapy: changing dietary concepts. In: Danowski TS, editor. Diabetes mellitus: diagnosis and treatment, vol. 1. New York: American Diabetes Association; 1964, p. 73–78.

[14] Jonckheer J, Demol J, Lanckmans K, Malbrain M, Spapen H, De Waele E. MECCIAS trial: metabolic consequences of continuous veno-venous hemofiltration on indirect calorimetry. Clin Nutr. 2020;39:3797–803. 32371095 10.1016/j.clnu.2020.04.017

[15] Levraut J, Garcia P, Giunti C, Ichai C, Bouregba M, Ciebiera JP, et al. The increase in CO_2_ production induced by NaHCO_3_ depends on blood albumin and hemoglobin concentrations. Intensive Care Med. 2000;26:558–64. 10923730 10.1007/s001340051204

[16] Bell M, Hertzberg D, Hansson F, Carlsson A, Berkius J, Vimlati L, et al. Modern CRRT systems are associated with lower risk of hypothermia. Sci Rep. 2024;14:23162. 39369021 10.1038/s41598-024-74977-2 PMC11455880

[17] New AM, Nystrom EM, Frazee E, Dillon JJ, Kashani KB, Miles JM. Continuous renal replacement therapy: a potential source of calories in the critically ill. Am J Clin Nutr. 2017;105:1559–63. 28468893 10.3945/ajcn.116.139014 PMC6546225

[18] Rogers AR, Jenkins B. Calorie provision from citrate anticoagulation in continuous renal replacement therapy in critical care. J Intensive Care Soc. 2021;22:183–6. 34422098 10.1177/1751143720937451 PMC8373286

[19] Fiaccadori E, Pistolesi V, Mariano F, Mancini E, Canepari G, Inguaggiato P, et al. Regional citrate anticoagulation for renal replacement therapies in patients with acute kidney injury: a position statement of the Work Group “Renal Replacement Therapies in Critically Ill Patients” of the Italian Society of Nephrology. J Nephrol. 2015;28:151–64. 25585821 10.1007/s40620-014-0160-2

[20] Oudemans-van Straaten HM, Ostermann M. Bench-to-bedside review: citrate for continuous renal replacement therapy, from science to practice. Crit Care. 2012;16:249. 23216871 10.1186/cc11645 PMC3672558

[21] Wechselberger S, Compton F, Schilling J. Impact of continuous veno-venous HemoDiALYsis with regional citrate anticoagulation on non-NUTRItional calorie balance in patients on the ICU-the NUTRI-DAY study. Nutrients. 2022;15:63. 36615721 10.3390/nu15010063 PMC9824471

[22] Schneider AG, Journois D, Rimmele T. Complications of regional citrate anticoagulation: accumulation or overload?. Crit Care. 2017;21:281. 29151020 10.1186/s13054-017-1880-1 PMC5694623

[23] Hu D, Sheeja Prabhakaran H, Zhang YY, Luo G, He W, Liou YC. Mitochondrial dysfunction in sepsis: mechanisms and therapeutic perspectives. Crit Care. 2024;28:292. 39227925 10.1186/s13054-024-05069-w PMC11373266

[24] Singer P, Blaser AR, Berger MM, Calder PC, Casaer M, Hiesmayr M, et al. ESPEN practical and partially revised guideline: clinical nutrition in the intensive care unit. Clin Nutr. 2023;42:1671–89. 37517372 10.1016/j.clnu.2023.07.011

[25] Nash DM, Przech S, Wald R, O’Reilly D. Systematic review and meta-analysis of renal replacement therapy modalities for acute kidney injury in the intensive care unit. J Crit Care. 2017;41:138–44. 28525779 10.1016/j.jcrc.2017.05.002

